# Enhancing Genomic Prediction Accuracy with a Single-Step Genomic Best Linear Unbiased Prediction Model Integrating Genome-Wide Association Study Results

**DOI:** 10.3390/ani15091268

**Published:** 2025-04-29

**Authors:** Zhixu Pang, Wannian Wang, Pu Huang, Hongzhi Zhang, Siying Zhang, Pengkun Yang, Liying Qiao, Jianhua Liu, Yangyang Pan, Kaijie Yang, Wenzhong Liu

**Affiliations:** College of Animal Science, Shanxi Agricultural University, Taigu, Jinzhong 030801, China; pang_z_x@163.com (Z.P.); wannian1876@163.com (W.W.); 18534272944@163.com (P.H.); zhanghongzhi336@163.com (H.Z.); zhangsy9526@163.com (S.Z.); ypk0412@163.com (P.Y.); liyingqiao1970@163.com (L.Q.); ljhbeth@163.com (J.L.); panyy@sxau.edu.cn (Y.P.); kjyang@sxau.edu.cn (K.Y.)

**Keywords:** genomic prediction, ssGBLUP, pseudo QTNs, weighted genomic relationship matrix, simulated dataset

## Abstract

Genomic selection (GS) is a genetic breeding method that uses genome-wide marker information to improve the accuracy of the prediction of complex traits. The single-step GBLUP (ssGBLUP) model, which integrates pedigree, phenotypic, and genomic data, has improved genomic prediction. However, ssGBLUP assumes that all markers contribute equally to genetic variance, which can limit its predictive accuracy, especially for traits controlled by major genes. To overcome this limitation, we integrate results from genome-wide association studies (GWAS) into an enhanced ssGBLUP framework, termed single-step genome-wide association assisted BLUP (ssGWABLUP). Our approach assigns differential weights to markers on the basis of their GWAS results, thereby increasing the contribution of effective markers while diminishing the influence of ineffective ones during the construction of the genomic relationship matrix. By incorporating pseudo quantitative trait nucleotides (pQTNs) as covariates, we aim to capture the effects of markers closely associated with major causal variants, leading to the development of the ssGWABLUP_pQTNs. Compared with weighted ssGBLUP (WssGBLUP), the ssGWABLUP model demonstrated superior accuracy and dispersion across different genetic architectures. We then compared the performance of our proposed ssGWABLUP_pQTNs model against both ssGBLUP and ssGWABLUP across various genetic scenarios. Our results demonstrate that ssGWABLUP_pQTNs outperforms other models in terms of prediction accuracy, particularly in scenarios with simpler genetic architectures. Additionally, evaluation using pig dataset confirmed the effectiveness of ssGWABLUP_pQTNs, highlighting its potential for practical breeding applications. The incorporation of pQTNs and a weighted genomic relationship matrix presents a promising and potentially scalable approach to further enhance genomic prediction, with potential implications for improving the accuracy of genomic selection in breeding programs.

## Simple Summary

Genomic selection (GS) is a powerful tool for improving the accuracy of genetic predictions in animal breeding. The single-step GBLUP (ssGBLUP) model integrates genomic, pedigree, and phenotypic data but assumes that all genetic markers contribute equally, which may limit its predictive power. To address this, we developed an enhanced model, ssGWABLUP, which incorporates genome-wide association study (GWAS) results to assign differential weights to markers. Furthermore, we introduced the ssGWABLUP_pQTNs model that integrates pseudo quantitative trait nucleotides (pQTNs) identified from GWAS into the weighted framework. Using both simulated and real datasets, we demonstrated that ssGWABLUP_pQTNs consistently outperforms ssGBLUP and other models, particularly for traits influenced by a small number of major genes. These findings suggest that integrating GWAS-derived information into ssGBLUP can enhance genomic prediction accuracy, providing a promising approach for improving genetic evaluations in livestock breeding programs.

## 1. Introduction

Genomic selection (GS) has emerged as a powerful tool for genetic improvement, particularly for animal and plant breeding. GS predicts genomic-estimated breeding values (GEBVs) using genome-wide marker information [1], instead of traditional pedigree data. Initially, GS methods were based on the best linear unbiased prediction (BLUP) model, which estimates breeding values using the pedigree-based relationship matrix (A). Genomic BLUP (GBLUP) is currently the most commonly used method in GS [2,3]. GBLUP estimates GEBV on the basis of genomic relationship matrix (G) [3]. This method has shown tremendous potential in various fields, including livestock production [4], crop breeding [5], and the study of complex human diseases [6,7].

Due to technical and economic constraints, not all individuals can be genotyped in practical applications. To address this issue, the single-step GBLUP (ssGBLUP) model was developed [8,9], which uses all phenotypic, pedigree, and genotypic information simultaneously, including phenotypic information on non-genotyped individuals. Therefore, in ssGBLUP, the relationship matrix H is constructed by combining the G and A matrices. This has significantly enhanced the accuracy of genomic predictions [10,11]. However, the accuracy of GS relies on several factors, including the size of the reference population [12], the relationship between training and validation populations [1], and the genetic architecture and heritability of the trait [13,14].

The GBLUP and ssGBLUP methods usually assume that marker effects follow a normal distribution, meaning that all markers contribute equally to the total genetic variance [3,15]. However, biologically, markers near major causal variants are expected to explain more variance than other markers [16], which may limit the prediction accuracy of GS. Assigning differential weights to markers to construct a weighted G matrix on the basis of their contribution to genetic variance has been shown to enhance the predictive ability of both GBLUP [17,18] and ssGBLUP [19,20]. In the weighted approach, SNPs with higher linkage disequilibrium to causal variants are assigned greater weights, reflecting their stronger influence on the genetic architecture of the trait [21]. To further refine this method, a uniform weight is used for all SNPs within predefined genomic windows [20], enabling consideration of the collective influence of SNPs within chromosomal regions. This window-based weighting approach has been shown to yield more accurate estimates of GEBV, particularly in weighted GBLUP models [16,22,23].

Genome-wide association studies (GWASs) are powerful tools for identifying QTLs associated with traits, and integrating GWAS results into GS models can improve predictive performance [16]. Furthermore, pseudo quantitative trait nucleotides (pQTNs), defined as SNPs significantly associated with the trait but not necessarily true causal variants, selected from GWAS results have been suggested as valuable covariates that can be incorporated into GBLUP to improve prediction accuracy [18,24,25,26]. Although such approaches have been applied to enhance GBLUP, their integration into ssGBLUP remains largely unexplored. The weighted ssGBLUP (WssGBLUP) model improves prediction accuracy by iteratively estimating SNP effects and assigning higher weights to markers with greater contribution to the trait. However, this iterative weighting process is computationally intensive, particularly for large-scale datasets. For traits controlled by a small number of major-effect genes, GWAS can reliably detect markers in strong linkage disequilibrium with causal variants [27], allowing the identified pQTNs to capture a substantial proportion of the genetic variance through their association with these functional loci [28]. To further improve the accuracy and efficiency of ssGBLUP, we propose a novel model that integrates GWAS results by combining pQTNs with a weighted G matrix. Specifically, we first select pQTNs from GWAS results and include them as covariates in the ssGBLUP model to better capture their contribution to the trait. Additionally, we use GWAS results to differentially weigh all the markers in the G matrix [16], assigning higher weights to markers with stronger associations and lower weights to those with weaker associations.

## 2. Materials and Methods

### 2.1. Simulated Data

The population and genomic architecture were simulated using QMSim [29] for 10 replicates. The historical population began with 10,000 individuals, gradually decreased to 500 by generation 1000, and then increased to 50,000 by generation 1100. The fluctuations in population size were employed to generate linkage disequilibrium consistent with a dairy cattle population. The full pedigree spanned 1110 generations, with the recent population (generations 1101–1110) derived from 50 male and 4000 female founders selected at generation 1100. The sire and dam replacement rates were 80% and 30%, respectively, with 10 generations of random mating. Each female had one offspring per generation, with equal probabilities for male or female offspring. Individuals were selected based on EBV calculated from BLUP. The simulated genome, modeled after the dairy cattle genome, consisting of 29 autosomes with a total length of 2319 cm. The genome comprises 50,000 biallelic SNPs. Both SNPs had an allele frequency of 0.5, with a mutation rate of $2.5\times 10^{-5}$ per locus per meiosis, and a recombination rate of 1 crossover per morgan per meiosis. In the last four generations, 50% of the individuals were randomly selected for genotyping. After simulating the entire dataset in QMSim, we generated 5% missing pedigrees. We designated the individuals from the last generation as the validation population, while all previous generations were used as the reference population.

In this study, we refer to simulated loci with direct effects on the phenotype as causal SNPs rather than QTLs to avoid confusion with traditionally mapped QTL regions identified via association studies. To comprehensively evaluate genomic prediction models across diverse genetic architectures, we simulated three sex-limited traits (h2 = 0.1) under four distinct genetic scenarios: (1) 10 causal SNPs, (2) 100 causal SNPs, (3) 500 causal SNPs, and (4) 10 large-effect causal SNPs and 500 small-effect causal SNPs. For Scenarios 1–3, causal SNP effects followed a gamma distribution (shape parameter α = 0.4). Scenario 4 explicitly modeled traits with both major and polygenic components by randomly selecting 10 SNPs as large-effect (sampled from N(0, 0.1)) and 500 SNPs as small-effect (sampled from N(0, 0.001)), thereby reflecting realistic architectures observed in complex traits. Post-simulation quality control excluded SNPs with minor allele frequency (MAF) < 0.05, genotype call rate (CR) < 0.95, or significant deviation from Hardy–Weinberg equilibrium (HWE; *p* < 0.001).

### 2.2. Real Data

In this study, a real pig dataset was used [30]. The dataset included pedigree information for 6473 individuals, among which 1247 were founders, spanning 17 generations. Genotypic data were available for 3534 individuals, obtained using the PorcineSNP60 chip (Illumina, San Diego, CA, USA). SNPs with an MAF < 0.05, call rate CR < 0.95, or HWE *p* value < 0.001 were excluded, resulting in a total of 33,735 SNPs retained for analysis. Phenotypic data and EBVs were available for five continuous traits with varying heritabilities (T1 = 0.07, T2 = 0.16, T3 = 0.38, T4 = 0.58, T5 = 0.62). Phenotypes were pre-adjusted for fixed effects or represented weighted progeny mean-corrected phenotypes. The EBVs were derived from the full PIC genetic evaluation.

### 2.3. Single-Step GBLUP Model with Pseudo QTNs and a Weighted G Matrix

We consider the following model:

y=Xb+Pq+Wu+e

Here, y is a vector with phenotypic values, b is a vector with fixed effects, with the corresponding incidence matrix X. The term P is the incidence matrix for pQTNs, which are derived from a selection procedure based on a multiple regression model, and q represents the vector of pQTN effects. The random additive genetic effects are represented by u, and e denotes the random residual effects. The variance structure of the model is assumed as follows:

$$Var\left(y\right)=V,Var\left(u\right)=H\sigma_{u}^{2},and\;Var\left(e\right)=R=I\sigma_{e}^{2},$$

where $V=H\sigma_{u}^{2}+R$, and $\sigma_{u}^{2}$ and $\sigma_{e}^{2}$ are the genetic and residual variances, respectively. The kinship matrix H is constructed as follows:

$$H=\left[\begin{array}{cc} A_{nn}+A_{ng}A_{gg}^{-1}\left(G_{\omega }-A_{gg}\right)A_{gg}^{-1}A_{gn} & A_{ng}A_{gg}^{-1}G_{\omega }\\ G_{\omega }A_{gg}^{-1}A_{gn} & G_{\omega } \end{array}\right]$$

where $G_{\omega }=0.95G+0.05A_{gg}$ [8], G is the genomic relationship matrix, A is the full pedigree-based relationship matrix, $A_{nn}$ refers to the pedigree-based relationship matrix for non-genotyped individuals, $A_{gg}$ is the matrix for genotyped individuals, and $A_{ng}$ represents the relationship matrix between non-genotyped and genotyped individuals. The inverse of H, denoted $H^{-1}$, is constructed as follows:

$$H^{-1}=A^{-1}+\left[\begin{array}{cc} 0 & 0\\ 0 & G_{\omega }^{-1}-A_{gg}^{-1} \end{array}\right]$$

The genomic relationship matrix G is defined as follows [3]:

$$G=\frac{ZDZ^{'}}{\sum_{j}2p_{j}\left(1-p_{j}\right)}$$

Here, Z is a matrix of centered genotypes, where $Z_{ij}=M_{ij}-2p_{j}$. The term $M_{ij}$ represents the genotype of the ith animal and the jth marker, which can be 0, 1, or 2, representing the genotypes $A_{1}A_{1}$, $A_{1}A_{2}$, or $A_{2}A_{2}$, respectively. The allele frequency of the jth marker is denoted by $p_{j}$, and D is a diagonal matrix of weights, where diagonal element $d_{j}$ is the weight assigned to the jth marker. If no weights are applied (i.e., all markers are weighted equally), an unweighted genomic relationship matrix $G_{u}$ is obtained by setting D=I, and assigning a weight of 1 to all markers.

The mixed model equations for the model are given by the following:

$$\left[\begin{array}{ccc} X^{'}R^{-1}X & X^{'}R^{-1}P & X^{'}R^{-1}W\\ P^{'}R^{-1}X & P^{'}R^{-1}P & P^{'}R^{-1}W\\ W^{'}R^{-1}X & W^{'}R^{-1}P & W^{'}R^{-1}W+H^{-1}\sigma_{g}^{-2} \end{array}\right]\left[\begin{array}{c} \hat{b}\\ \hat{q}\\ \hat{u} \end{array}\right]=\left[\begin{array}{c} X^{'}R^{-1}y\\ P^{'}R^{-1}y\\ W^{'}R^{-1}y \end{array}\right]$$

After the mixed model equations are solved, the estimates of the breeding values ($\hat{g}$) are obtained by summing the estimated effects of the fixed and random components in the model as follows:

$$\hat{g}=P\hat{q}+\hat{u}$$

### 2.4. Genome-Wide Association Study (GWAS)

A GWAS is a powerful method for identifying candidate genes that contribute to complex traits. The classical GWAS method, EMMAX [31], models the effect of the jth marker as follows:

$$y=Xb+M_{j}a_{j}+u+e$$

where $a_{j}$ is the allele substitution effect of the jth marker, and u denotes the random effect of polygenes, which is assumed to follow a normal distribution $u\sim N(0,G_{u}\sigma_{u}^{2})$. To test the effect of the marker, a hypothesis test is used with the statistic ${\hat{a}}_{j}/se_{j}$, where ${\hat{a}}_{j}$ is the estimate of the jth marker effect, and $se_{j}$ is the standard error of the marker effect estimate, calculated as $se_{j}={\left(M_{j}^{'}V^{-1}M_{j}\right)}^{-\frac{1}{2}}$.

### 2.5. Single Step Genome-Wide Association Assisted BLUP (ssGWABLUP)

The marker weights in the G matrix were adjusted on the basis of the results of GWAS [16]. The log-likelihood ratio (LR) for the jth marker is given by $LR_{j}=\frac{1}{2}{\left({\hat{a}}_{j}/se_{j}\right)}^{2}$. To reduce noise, a smoothing approach is applied by taking the moving average of the LR for the jth marker and its surrounding 20 markers. Posterior probabilities (PPs) for each marker are then computed using the smoothed LR values:

$$PP_{j}=\pi e^{LR_{j}}/\;[\pi e^{LR_{j}}+(1-\pi )]$$

where π is the prior probability that a marker has an effect, typically set to a small value (0.001) [16]. These PP values are used as marker weights to construct the weighted genomic relationship matrix. The H matrix constructed using the weighted G matrix is referred to as $H_{w}$.

### 2.6. Pseudo QTNs Selection

First, the markers were ranked by *p* value, and markers were sequentially selected, ensuring that each new marker had a linkage disequilibrium ($r^{2}$) below 0.3 with the previously selected markers, until 15 markers were chosen. The reference population was then divided into five folds for cross-validation to assess the predictive accuracy. Each selected marker was subsequently incorporated into the model as a covariate, and if the new model’s accuracy improved by ≥0.001, the marker was added to the pQTLs. This process was repeated until all 15 markers were evaluated, and the pQTNs that effectively improved the model were selected. The validated pQTNs are subsequently used to construct the $P_{g}$ matrix for genotype animals. Finally, we impute pQTNs for non-genotype animals ${\hat{P}}_{n}=A_{ng}A_{gg}^{-1}P_{g}$. In this study, the models incorporating pQTNs are denoted with the suffix “pQTNs”.

This study uses two elements: the presence of pQTNs (whether they exist), and the different possible forms of D (where D=I or $D=diag(PP_{j})$), resulting in four models: ssGBLUP, ssGBLUP_pQTNs, ssGWABLUP, and ssGWABLUP_pQTNs (Table 1).

### 2.7. Benchmarking

In addition to the proposed models, we included the WssGBLUP [20,21] for comparison. The model iteratively re-estimates SNP effects using GEBVs and updates marker weights to construct a weighted genomic relationship matrix. This approach allows markers with greater influence on the trait to contribute more to the genomic prediction. In this study, we implemented WssGBLUP using the blupf90 family (2022-05-27) software [9,32]. Specifically, we used blupf90 to estimate GEBVs, postGSf90 to backsolve SNP effects from GEBVs, and nonlinear A [3] to calculate SNP weights. According to the findings of previous studies [21,33], prediction accuracy peaks at the second iteration before slightly declining; therefore, all reported results are based on the second iteration.

To provide an additional comparison with our proposed model on the pig dataset, we implemented a single-step Bayesian regression approach using the hibayes package [34]. This method extends the BayesR model [35,36] to accommodate both genotyped and non-genotyped individuals by jointly modeling pedigree, genotypes, and phenotypes. Single-step BayesR (ssBayesR) assumes that SNP effects follow a mixture of four normal distributions with different variances, including a zero-effect distribution. Specifically, SNP effects are sampled from a prior distribution:

$$\begin{array}{l} \;p\left(g_{j}|\pi ,\sigma_{g}^{2}\right)=\pi_{1}\times N\left(0,0\times \sigma_{g}^{2}\right)+\pi_{2}\times N\left(0,0.01\times \sigma_{g}^{2}\right)\\ +\pi_{3}\times N\left(0,0.001\times \sigma_{g}^{2}\right)+\pi_{4}\times N\left(0,0.0001\times \sigma_{g}^{2}\right) \end{array}$$

where $\pi_{1}$, $\pi_{2}$, $\pi_{3}$, and $\pi_{4}$ are the proportions of markers assigned to each distribution and $\sigma_{g}^{2}$ is the total additive genetic variance. The analysis was conducted using the ssbrm () function in the hibayes package [34]. A Gibbs sampling algorithm was used with 50,000 iterations, including 10,000 burn-in and a thinning interval of 10.

### 2.8. Validation of Genomic Predictions

To validate the genomic predictions, three key validation metrics were used: correlation, bias, and dispersion. These measures were applied to compare the different evaluation models. The Pearson correlation coefficient was calculated between the true breeding value (TBV) and EBV, providing an assessment of the linear relationship between them. Bias was defined as the difference between the average EBV and TBV, expressed as $Bias=\bar{GEBV}-\bar{TBV}$. Dispersion was evaluated by the regression slope of the EBV on the TBV, represented as $Dispersion=\frac{Cov\left(GEBV,TBV\right)}{Var\left(TBV\right)}$, which indicates how well the spread of EBV matches that of TBV. All metrics were calculated based on the youngest animals born in the final simulated generation to ensure a consistent and fair comparison across models. For real traits, genomic predictions were evaluated using phenotypic data. A ten-fold cross-validation procedure was implemented to compare the predictive performance of different methods. The average of the ten Pearson correlation coefficients between GEBV and EBV (come from the full PIC genetic evaluation) in the validation sets was used as the measure of prediction accuracy.

## 3. Results

### 3.1. Comparison of Performance Between ssGWABLUP and WssGBLUP

**Figure 1:**
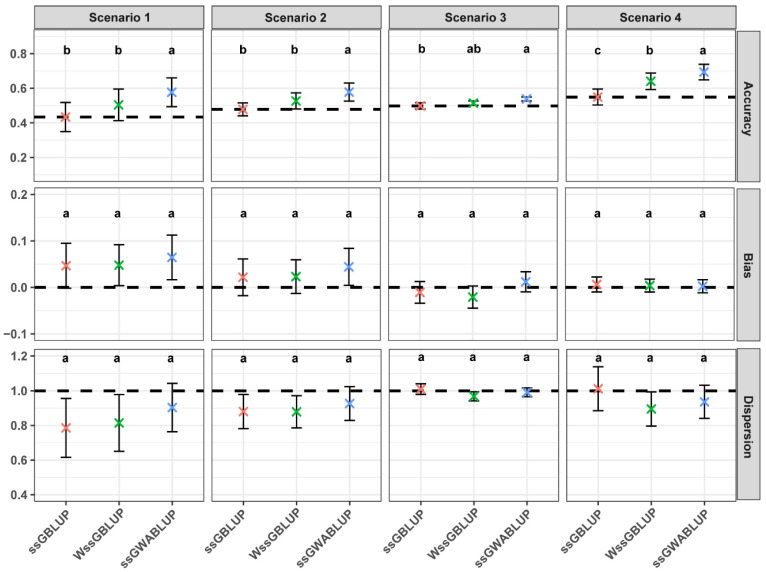
Comparative performance (correlation, bias, and dispersion) of ssGBLUP, ssGWABLUP and WssGBLUP models across different scenarios. The error bars in the plot show standard deviation and the “×” show the means over 10 repetitions. Different letters above the bars denote statistically significant differences between models (*p* < 0.05).

Across the 10 simulation replicates, ssGWABLUP consistently demonstrated enhanced performance in terms of correlation, bias, and dispersion across all the scenarios (Figure 1). In all cases, ssGWABLUP outperformed WssGBLUP by achieving higher correlation, slightly greater bias, and comparable dispersion. This trend was observed across the different scenarios, with ssGWABLUP maintaining better overall prediction accuracy and stability.

The computational efficiency of ssGWABLUP was notably superior to that of WssGBLUP across all scenarios, as it consistently required less time and memory (Table 2). This demonstrates that ssGWABLUP not only improves predictive performance but also offers better computational efficiency, making it more scalable and practical for larger datasets.

### 3.2. Impact of Genetic Complexity on pQTN Selection and Effectiveness

**Figure 2:**
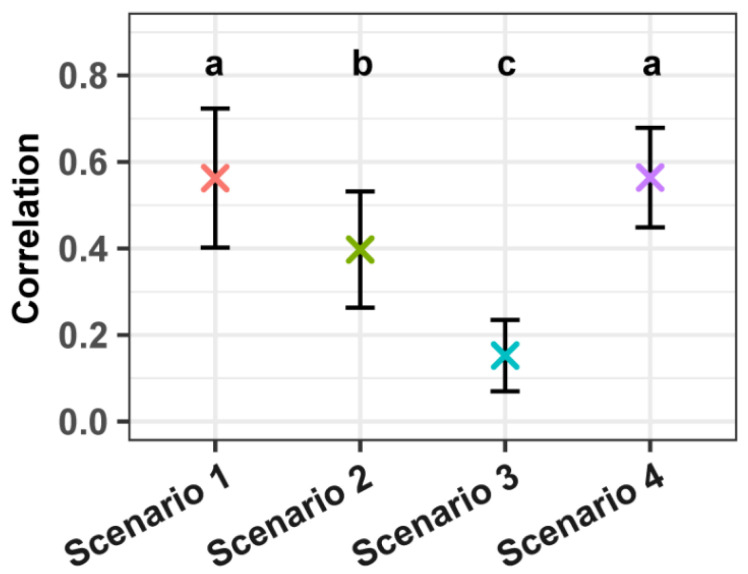
Correlation between pQTN effects and TBV across different scenarios. The error bars in the plot show standard deviation and the “×” show the means over 10 repetitions. Different letters above the bars denote statistically significant differences between scenarios (*p* < 0.05).

The integration of pQTNs into the ssGBLUP model demonstrated a notable impact across all simulated scenarios. In scenario 1, the average number of selected pQTNs was highest. As the number of causal SNPs increased to 100 and 500 in scenarios 2 and 3, respectively, the average number of selected pQTNs decreased, indicating a reduced capacity for pQTN selection in more complex genetic architectures (Table 3). Additionally, the correlation between pQTN effects and TBV followed a similar pattern, with the strongest correlation observed in scenario 1 and progressively weaker correlations as the number of causal SNPs increased (Figure 2). These results reflect the diminishing influence of pQTNs on prediction accuracy as genetic complexity increases. Notably, scenario 4, which combined 10 large-effect with 500 small-effect causal SNPs, presented a unique pattern. Despite its complexity, this scenario maintained a relatively high average number of selected pQTNs and a strong correlation between pQTN effects and TBV, comparable to scenario 1. This suggests that the presence of large-effect causal SNPs can enhance the detection and predictive power of pQTNs, even amidst a polygenic background. Therefore, integrating pQTNs into ssGBLUP models may be particularly beneficial in traits where major genes coexist with numerous minor ones, as it allows for effective capture of significant genetic signals contributing to trait variation.

### 3.3. Performance Evaluation of the ssGWABLUP_QTNs

**Figure 3:**
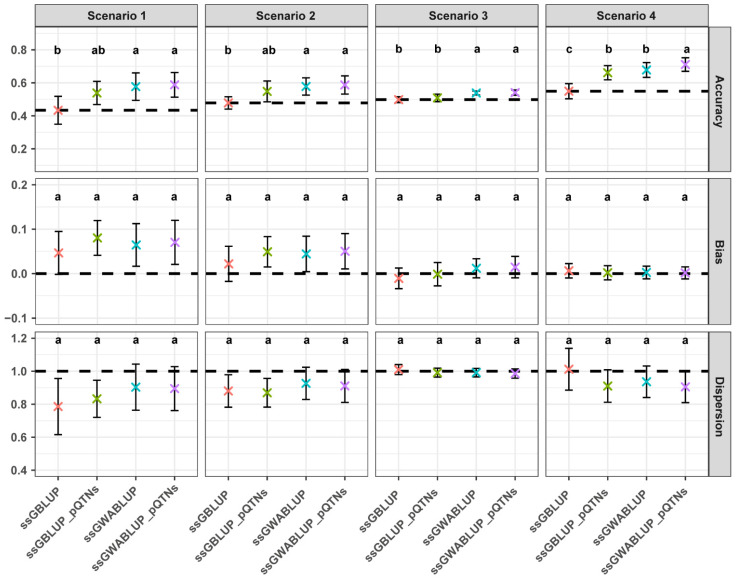
Comparative performance (correlation, bias, and dispersion) of ssGBLUP, ssGBLUP_pQTNs, ssGWABLUP, and ssGWABLUP_pQTNs models across different scenarios. The error bars in the plot show standard deviation and the “×” show the means over 10 repetitions. Different letters above the bars denote statistically significant differences between models (*p* < 0.05).

The performance evaluation of the ssGWABLUP_pQTNs model across various scenarios demonstrated improvements in prediction accuracy compared with the baseline ssGBLUP and ssGWABLUP models (Figure 3). Across scenarios, the ssGWABLUP_pQTNs model consistently showed higher accuracy than other methods, reduced bias, and well-maintained dispersion. This improvement is most evident in the scenario 1 and scenario 4, where the model achieved both higher accuracy and a favorable balance in bias and dispersion. Notably, in scenario 4, which involves a mixture of large- and small-effect causal SNPs, the ssGWABLUP_pQTNs model attained the highest average accuracy among all scenarios, along with minimal bias and controlled dispersion, highlighting its robustness in capturing genetic signals in mixed-effect architectures. Even in polygenic scenarios, such as the scenario 3, the model exhibited superior performance, maintaining better accuracy while controlling bias and dispersion effectively. These results indicate that the incorporation of pQTNs into the ssGWABLUP framework enhances the model’s ability to predict genetic values across varying genetic architectures.

### 3.4. Performance on Pig Dataset

The predictive performance of the five models was evaluated using real pig trait data, and the results are summarized in Table 4. Overall, the proposed ssGWABLUP_pQTNs model consistently achieved the highest prediction accuracy across all traits. It was followed by ssGWABLUP, ssGBLUP_pQTNs, ssGBLUP, and the ssBayesR. The improvements in accuracy were particularly notable for T3 and T5, where the incorporation of pQTNs and marker weighting led to observable gains. The ssGWABLUP model outperformed ssGBLUP across all traits, indicating the advantage of assigning differential weights to markers. Similarly, integrating pQTNs into both models further enhanced predictive performance. The ssBayesR model also demonstrated reasonable performance, but its accuracy was generally lower than that of the ssGWABLUP_pQTNs model. These results demonstrate the effectiveness of incorporating GWAS-derived pQTNs and marker weighting in genomic prediction, particularly for traits with moderate to high heritability.

## 4. Discussion

This study introduced an improved ssGBLUP model that integrates pQTNs derived from GWAS with a weighted G matrix to increase the accuracy of GS. The model performance was evaluated using simulated genomic data, and the results demonstrated superior predictive ability compared with both the traditional ssGBLUP and WssGBLUP models across different genetic architectures.

Comparative analysis of ssGWABLUP and WssGBLUP revealed that ssGWABLUP consistently outperformed WssGBLUP across different scenarios, particularly for traits controlled by large-effect causal SNPs (scenarios 1 and 4). The enhanced correlation observed in ssGWABLUP is indicative of its superior predictive ability, particularly when the number of causal SNPs was small. Previous studies have similarly highlighted the benefit of using GWAS results to differentially weigh all the SNPs in a weighted GBLUP genomic prediction analysis [16,37]. As the genetic complexity increased, reflected by a higher number of causal SNPs, the performance advantage between ssGWABLUP and WssGBLUP diminished. While ssGWABLUP maintained its advantage over WssGBLUP, the magnitude of improvement relative to the traditional ssGBLUP model decreased. This suggests that as the number of causal SNPs increases, the ability of both models to capture the full scope of genetic variance becomes limited [20,37]. To address this limitation, the ssGWABLUP_pQTNs model was developed by further integrating pQTNs into the ssGWABLUP framework. Building upon ssGWABLUP, the ssGWABLUP_pQTNs model further improved prediction by explicitly incorporating pQTNs. In the simulation results, ssGWABLUP_pQTNs outperformed all other models in terms of prediction accuracy, reduced bias, and stable dispersion, particularly under scenarios 1 and 4. In the pig dataset, the genomic prediction results for five economic traits further validated the advantage of ssGWABLUP_pQTNs (Table 3). Compared with ssGBLUP, ssGWABLUP exhibited higher prediction accuracy across all traits, while ssGWABLUP_pQTNs outperformed all other methods in every trait, further supporting the conclusions drawn from the simulation data.

Across different simulation scenarios, a clear trend emerged in the number of selected pQTNs and their correlation with TBV. In scenario 1, which involved a small number of causal SNPs with large effects, a greater number of pQTNs were identified, and these showed the highest correlations with TBV. This observation is consistent with previous studies suggesting that loci with large genetic effects are more readily detected through GWAS [38,39]. In scenario 2, where the number of causal SNPs increased moderately, the number of identified pQTNs and their correlation with TBV declined, reflecting the increased difficulty in detecting variants of moderate effect sizes. Scenario 3, characterized by a high number of causal SNPs, both the number of selected pQTNs and their correlation with TBV were markedly reduced, indicating the limited capacity of GWAS to detect small-effect loci in highly polygenic traits. These results align with prior findings that as the number of causal variants increases, the signal attributable to any single marker becomes diluted, making accurate detection more difficult [14,40,41]. Interestingly, scenario 4, which involved a mixture of large- and small-effect causal SNPs, demonstrated intermediate performance. Although the number of pQTNs identified was lower than in scenario 1, those that were detected maintained relatively high correlations with TBV, suggesting that the model remains effective at capturing major-effect loci even in mixed-effect genetic architectures. Collectively, these results indicate that the ability to detect informative pQTNs is highly dependent on the underlying genetic architecture. Simpler architectures with fewer, larger-effect causal SNPs allow for more accurate identification of genetic signals, whereas increased genetic complexity reduces the power of GWAS to isolate informative markers, consistent with observations from previous research [40,42,43]. While the current study primarily focused on evaluating the predictive accuracy of the selected pQTNs across different genetic architectures, an important direction for future work is to assess the biological relevance of these markers. Specifically, evaluating whether the pQTNs are located in or near known functional genes or regulatory elements associated with the trait could provide additional support for their role in genetic architecture.

Although ssGWABLUP had already assigned higher weights to major effect markers, the ssGWABLUP_pQTNs model further improved prediction performance by integrating pQTNs with a weighted G matrix. This enhancement was particularly evident under simpler genetic architectures, such as scenario 1. In such cases, pQTNs derived from GWAS more precisely captured the effects of major genes, thereby reinforcing the predictive signal. Meanwhile, the weighted G matrix facilitated the optimal integration of information from the remaining markers, allowing the model to account for additional sources of genetic variance. In the more complex architecture of scenario 4, relying solely on pQTNs may overlook the contributions of small-effect causal SNPs, while depending only on the weighted G matrix may dilute the effects of major causal SNPs. The ssGWABLUP_pQTNs model, by combining both approaches, leverages the strong signals identified by GWAS while maintaining coverage of weaker signals, creating a complementary effect that collectively enhances prediction accuracy. Overall, this integration strategy effectively balances the strengths of GWAS-based variant selection and genome-wide marker weighting, enhancing the robustness and adaptability of the model across diverse genetic architectures.

While the findings of this study highlight the advantages of integrating GWAS-derived pQTNs into ssGBLUP, several considerations should be noted regarding its application in practical breeding programs. The ssGWABLUP_pQTNs model is particularly advantageous for traits controlled by major genes, where pQTNs can effectively capture the most relevant genetic signals. This suggests potential applications in breeding programs targeting economically important traits that are influenced by a small number of genes with large effects, such as milk yield [44] and carcass quality [45] in livestock. However, for complex traits regulated by numerous small-effect loci, the advantage of incorporating pQTNs may be less pronounced, as the model’s ability to fully capture the genetic variance may be constrained. In addition, the model is sensitive to the prior probability π used in marker weighting, and the inclusion of too many pQTNs may lead to overfitting. Moreover, the effectiveness of the model relies heavily on the quality of GWAS results, which may be affected by false positives depending on the statistical methods and thresholds applied. This suggests that, in practical applications, further optimization of prior probability settings is necessary [16], along with enhancing signal quality through meta-analysis [46] of GWAS across populations. Furthermore, the stability of selected pQTNs may depend on the specific GWAS method used, as different statistical approaches and significance thresholds can influence marker selection. This highlights the importance of robust and consistent pQTN identification methods to ensure the reproducibility and reliability of predictions. In terms of computational efficiency, ssGWABLUP presented lower computational time and memory consumption than WssGBLUP did. This advantage indicates better scalability and practicality when dealing with large datasets. However, the current study focused only on simulated populations and a single-population dataset, which limits the assessment of the model’s generalizability. In animal breeding, different breeds or species often exhibit distinct LD structures and genetic architectures [5,47,48]. The performance of ssGWABLUP_pQTNs in such populations remains unclear and warrants further investigation. Future work should explore its robustness across diverse genetic backgrounds to confirm its broader applicability.

## 5. Conclusions

In conclusion, this study demonstrated that the ssGWABLUP_pQTNs model provides significant improvements in predictive accuracy, bias, and dispersion compared with both the ssGBLUP and WssGBLUP models, particularly in simpler genetic architectures. While the improvements were more significant for traits influenced by major genes, the model also maintained advantages in traits with a polygenic architecture. The integration of pQTNs, which were selected on the basis of GWAS results, into the ssGBLUP framework was shown to enhance the model’s ability to predict genetic values across different QTL scenarios. However, as genetic complexity increases, the effectiveness of pQTN selection diminishes, highlighting the challenges posed by polygenic traits. Despite these limitations, the ssGWABLUP_pQTNs model holds promise for practical implementation in breeding programs, especially for traits influenced by a small number of large-effect QTLs. Its relatively low computational cost also supports its applicability in large-scale selection programs. Nevertheless, additional validation is needed in more diverse breeding contexts, such as multi-breed populations, crossbred animals, or populations with limited genotyping resources. Future studies should also focus on integrating biological annotations (e.g., gene function or expression profiles), reported QTLs, and coding information into the model to further enhance its robustness and biological relevance.

## Figures and Tables

**Table 1:** Comparison of ssGBLUP-based models.

Model	Equation	Explanation
ssGBLUP	$y=Xb+Wu+e,u\sim N(0,H\sigma_{u}^{2})$	Standard single-step GBLUP model using a $G_{u}$ matrix.
ssGBLUP_pQTNs	$y=Xb+Pq+Wu+e,u\sim N(0,H\sigma_{u}^{2})$	Extends ssGBLUP by including pQTNs as fixed covariates.
ssGWABLUP	$y=Xb+Wu+e,u\sim N(0,H_{w}\sigma_{u}^{2})$	Incorporates SNP weights derived from GWAS results into the G matrix.
ssGWABLUP_pQTNs	$y=Xb+Pq+Wu+e,u\sim N(0,H_{w}\sigma_{u}^{2})$	Combines marker weighting from GWAS and inclusion of pQTNs as covariates.

y is the phenotypic values, Xb represents fixed effects, Wu denotes the random genetic effects, and e is the residual error. Pq represents the fixed effects of selected pQTNs. H is the combined relationship matrix based on the unweighted genomic relationship matrix $G_{u}$, and $H_{w}$ is the modified relationship matrix constructed using a GWAS-based weighted G matrix.

**Table 2:** Computational time and memory usage for WssGBLUP and ssGWABLUP across different scenarios.

Scenario	Time (s)		Memory (Gb)	
	WssGBLUP	ssGWABLUP	WssGBLUP	ssGWABLUP
Scenario 1	50.25	9.66	4.32	2.36
Scenario 2	46.67	10.58	4.32	2.34
Scenario 3	54.28	12.08	4.32	2.35
Scenario 4	43.30	12.10	4.32	2.37

**Table 3:** Number of selected pQTNs across scenarios.

Scenario	Mean	SD
Scenario 1	6.90	1.73
Scenario 2	5.30	1.25
Scenario 3	2.78	1.09
Scenario 4	5.90	0.74

**Table 4:** The mean and standard deviation of prediction accuracies for ssGBLUP, ssGBLUP_pQTNs, ssGWABLUP, ssGWABLUP_pQTNs, and ssBayesR models for real traits in pig dataset.

Traits	ssGBLUP	ssGBLUP_pQTNs	ssGWABLUP	ssGWABLUP_pQTNs	ssBayesR
T1	0.283 ± 0.047 ^b^	0.288 ± 0.049 ^ab^	0.291 ± 0.049 ^a^	0.297 ± 0.048 ^a^	0.269 ± 0.040 ^c^
T2	0.717 ± 0.040 ^c^	0.728 ± 0.041 ^b^	0.730 ± 0.038 ^ab^	0.741 ± 0.034 ^a^	0.727 ± 0.040 ^b^
T3	0.545 ± 0.025 ^b^	0.581 ± 0.029 ^a^	0.579 ± 0.026 ^a^	0.587 ± 0.028 ^a^	0.585 ± 0.029 ^a^
T4	0.603 ± 0.048 ^b^	0.613 ± 0.056 ^b^	0.628 ± 0.048 ^a^	0.630 ± 0.055 ^a^	0.625 ± 0.047 ^a^
T5	0.535 ± 0.033 ^c^	0.549 ± 0.036 ^b^	0.550 ± 0.035 ^b^	0.573 ± 0.034 ^a^	0.569 ± 0.034 ^a^

Different letters above the bars denote statistically significant differences between models (*p* < 0.05).

## Data Availability

The pig dataset http://www.g3journal.org/lookup/suppl/doi:10.1534/g3.111.001453/-/DC1 (accessed on 1 February 2025). Code are available at: https://github.com/ZhixuPang/ssGBLUP_pQTNs.
